# A Broad Role for Cysteines in Bovine Antibody Diversity

**DOI:** 10.4049/immunohorizons.1900058

**Published:** 2019-10-16

**Authors:** Jeremy K. Haakenson, Thaddeus C. Deiss, Gabrielle F. Warner, Waithaka Mwangi, Michael F. Criscitiello, Vaughn V. Smider

**Affiliations:** * Applied Biomedical Science Institute, San Diego, CA 92127; † Department of Molecular Medicine, The Scripps Research Institute, La Jolla, CA 92037; ‡ College of Veterinary Medicine and Biomedical Sciences, Texas A&M University, College Station, TX 77843; § College of Veterinary Medicine, Kansas State University, Manhattan, KS 66506

## Abstract

Ab diversity in most vertebrates results from the assortment of amino acid side chains on CDR loops formed through V(D)J recombination. Cows (*Bos taurus*) have a low combinatorial diversity potential because of a small number of highly homologous V, D, and J gene segments. Despite this, a subset of the Ab repertoire (~10%) contains exceptionally long CDR H chain (HC) 3 (H3) regions with a rich diversity of cysteines and disulfide-bonded loops that diversify through a single V-D-J recombination event followed by massive somatic hypermutation. However, the much larger portion of the repertoire, encoding shorter CDR H3s, has not been examined in detail. Analysis of germline gene segments reveals noncanonical cysteines in the HC V regions and significant cysteine content in the HC D regions. Deep sequencing analysis of naturally occurring shorter CDR H3 (<40 aa) Ab genes shows that HC V and HC D regions preferentially combine to form a functional gene with an even number of total cysteines in the final V region, suggesting that disulfide bonds contribute to diversity not only in ultralong CDR H3 bovine Abs but in shorter CDR H3 bovine Abs as well. In addition to germline “hard-coded” cysteines, the bovine Ab repertoire can produce additional cysteine codons through somatic hypermutation, further diversifying the repertoire. Given the limited combinatorial diversity at the bovine Ig loci, this helps to explain how diversity is created in shorter CDR H3 Abs and potentially provides novel structural paratopes in bovine Ab combining sites.

## INTRODUCTION

Abs make up the major serum component of the vertebrate adaptive immune system, with a diverse Ig repertoire being necessary to neutralize invading microorganisms. Ab structural diversity is created through four processes: 1) V(D)J recombination, 2) its associated end-joining reaction to create junctional diversity, 3) pairing of H chains (HC) and L chains (LC), and 4) activation-induced cytidine deaminase (AID)–mediated somatic hypermutation (SHM). A feature unique to cows is the long length of CDR H chain 3 (H3) regions, with ~10% of the repertoire having an ultralong CDR H3 of between 40 and 70 aa (1-4). Even the shorter, non-ultralong component has lengths averaging well over 20 residues. These are quite long compared with other species whose CDR H3 lengths often average far fewer than 15 residues. Although cows contain 10 unique highly homologous, functional HC V (VH), 10 HC D (DH), and 4 HC J (JH) regions (5), only one VH, DH, and JH region appear to be used to form ultralong CDR H3 Abs (6). Shorter CDR H3 bovine Abs, in contrast, seem to use the full range of functional VH and DH genes for V(D)J recombination. Despite this, the combinatorial potential of the entire bovine Ab repertoire is still limited compared with humans, who have 55 VH, 23DH, and 6 JH gene segments (7). In this regard, there are 7590 possible VDJ combinations in humans compared with only ~400 in cows. Therefore, cows may rely on additional mechanisms for creating a structurally diverse Ab repertoire prior to Ag exposure.

AID is known to play a crucial role in diversifying Ab Ag binding sites. In humans and mice, this is thought to occur alter Ag exposure (8). However, in cows, SHM also contributes to Ab diversity prior to Ag exposure (2,9,10). V regions are preferentially targeted by AID (11), and this process appears to be very important in creating diversity in preimmune bovine ultralong CDR H3 Abs. In ultralong CDR H3 Abs, which contain a “stalk and knob” structure within the CDR H3, diversity is created by massive SHM on the single V-D-J unit, which not only produces amino acid changes but also diversifies disulfide bond patterns through cysteine mutation (2). The single DH region used in ultralong bovine Abs (IGHD8-2) directly encodes four cysteines in addition to many amino acids that are readily mutated to cysteine by AID (2), allowing different disulfide bond patterns to be formed, which creates a remarkable source of structural diversity for these Abs (2, 6).

Cysteine is unique among the 20 aa in that its sulfhydryl side chain, when forming disulfide bonds, can dramatically impact the secondary and tertiary structure of a protein. Disulfide bonds enable loop formation within a polypeptide chain, can stabilize domains, and when altered, can result in significant changes to the topology of the protein (12-14). The “knob” component of ultralong CDR H3 Abs, as a distinct and often cysteine-rich domain, can diversify its cysteine content to alter loop patterns and secondary structure. The potential of Abs with shorter CDR loops to use cysteine diversifications, either within CDR loops, between CDRs, or between framework regions (FW) and CDRs, to diversify the repertoire may also be an important diversity-generating mechanism in cows or other species.

Given the low diversity potential of the bovine germline repertoire, we investigated the possible role of cysteines in creating diversity in the entire bovine Ab system. Remarkably, cysteines are unexpectedly heavily encoded in VH and DH germline regions of cows, which results in a diverse mature repertoire with great potential for alternative and diverse disulfide-bonded loops.

## MATERIALS AND METHODS

### Germline sequences

Bovine VH sequences were from Deiss et al. (5), and bovine DH sequences, which are available in the International Immunogenetics Information System (IMGT) (7), were from Ma et al. (15). Bovine LC V (VL) sequences were obtained from the Bovine Genome Database (16). Germline DH sequences for human (*Homo sapiens*), mouse (*Mus musculus*), rat (*Rattus norvegicus*), alpaca (*Vicugna pacos*), dog (*Canis lupus*) rabbit (*Oryctolagus cuniculus*), sheep (*Ovis aries*), pig (*Sus scrofa*), platypus (*Ornithorhynchus anatinus*), and chicken (*Gallus gallus*) were obtained from IMGT.

### Structural modeling

The crystal structure of a Fab fragment of BLV1H12 (Protein Data Bank [PDB]: 4K3D), an ultralong bovine Ab, was downloaded from the PDB (www.rcsb.org). All modeling was then performed using PyMOL.

### Collection of blood samples, isolation of PBMCs and RNA, and synthesis of 5' RACE libraries

Tissues (blood, Peyer patch, spleen, and bone marrow) were derived from two adult cows housed at Texas A&M University Veterinary Medical Park under approved Animal Use Protocol 2015-0078. PBMCs were isolated from blood with lymphocyte separation media (Mediatech, Tewksbury, MA), and total RNA extraction was performed on the isolated PBMCs with the RNeasy Mini Kit (QIAGEN, Valencia, CA) as previously described (17). Isolated RNA was used as the template for the synthesis of 5′ RACE libraries with the GeneRacer Kit (Invitrogen, Carlsbad, CA), as previously described (18). An equal mix of oligoDT and random hexamer primers was used to prime the reaction.

### Amplification of IGH transcripts and Pacific Biosciences deep sequencing

The cDNA template produced in the 5′ RACE libraries was used as a template for PCR using the Phusion High-Fidelity Polymerase (New England BioLabs, Ipswich, MA). Products of 450–650 bp were visualized on an agarose gel and extracted. Pooled samples were sent to the Duke University Center for Genomic and Computational Biology Core Center for Pacific Biosciences library preparation and sequencing. Circular consensus sequences (sequences in which the Pacific Biosciences polymerase circled the insert at least three times) were returned in fastq format. The resulting fastq files were imported into Geneious V9 (Biomatters, Auckland, New Zealand), in which barcoded primers were used to demultiplex the samples. Finally, the sequences were quality filtered (*Q* > 20), and homopolymer runs were corrected using the ACACIA program (19).

Sequences were analyzed using the R Studio software program with the Bioconductor suite.

### Collection of blood samples, isolation of PBMCs and RNA, amplification of Igλ transcripts, and sequencing of LC genes

Blood was derived from an adult cow (Institutional Animal Care and Use Committee protocol no. 13-0010; ProSci). PBMCs were isolated from blood using Histopaque Hybri-Max (Sigma-Aldrich, St. Louis, MO), and total RNA extraction was performed on the isolated PBMCs with the RNeasy Mini Kit (QIAGEN), according to the manufacturer’s protocol. Isolated RNA was reverse transcribed into cDNA, and bovine Igl genes were amplified in one step using the One Step RT-PCR Kit (QIAGEN) with the following primers: 5′-GCTGGTCGCTCTCTGCACAGGATCCTGGGC-3′ and 5′-CTCCTCCKTGGAGGGCGGGAACAGGGTGA-3′, where K is a mixed base composed of G and T. The resulting products were then cloned into the pFuse vector (InvivoGen, San Diego, CA) and sent to GENEWIZ (La Jolla, CA) for sequencing.

## RESULTS

### Germline-encoded cysteines

**FIGURE 1.**
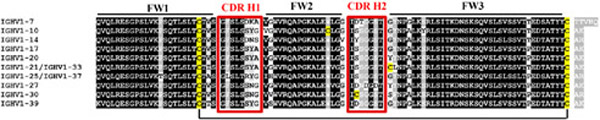
Functional bovine germline VH regions encode noncanonical cysteines. CDR H1 and CDR H2 are boxed in red, and the intervening FW are indicated. Cysteines are highlighted in yellow. Cys22 and Cys95 are completely conserved and form a required disulfide bond in the center of the Ig fold. Note the free cysteines in IGHV1-10, IGHV1-21/IGHV1-33, and IGHV1-30 (IGHV1-21 and IGHV1-33 and IGHV1-25 and IGHV1-37 are functional germline VH regions with identical amino acid sequences).

Cysteines play a major role in generating the structural diversity of the ultralong CDR H3 subset of bovine Abs (2). However, this subset only represents a small portion (~10%) of the repertoire. Because the remaining shorter CDR H3 component (~90%) uses only a few highly homologous VH regions, the overall repertoire diversity of cows may be severely limited compared with other species. Interestingly, in the course of analyzing bovine Ab HC sequences, we observed that many shorter CDR H3s contained an odd number of cysteines, which, in the absence of a disulfide bonding partner, would be left unpaired. This led us to hypothesize that these potentially unpaired cysteines in CDR H3 might disulfide bond with cysteines in the VH regions outside of CDR H3. Of particular interest was whether cysteines may be “hard-coded” in the germline, such that new disulfide bonds might be formed within CDRs, between CDRs, or between FW and CDRs. All Ig domains contain a completely conserved disulfide bond (i.e., two cysteines) that is required for its structural integrity, so any additional cysteines in the VH regions would be noncanonical (15). To determine if germline bovine VH regions contain potentially unpaired cysteines that might form disulfide bonds with unpaired cysteines in CDR H3s, we aligned the amino acid sequences of all of the bovine germline VH regions, which revealed that 3 out of 10 (30%) functional VH regions contain a noncanonical cysteine in or near CDR HC 2 (H2) (Fig. 1). Specifically, the highly used IGHV1-10 gene has a unique cysteine 4 aa upstream of CDR H2 in FW2, IGHV1-21/IGHV1-33 has a cysteine immediately after CDR H2 in FW3, and the second amino acid in CDR H2 of IGHV1-30 is a noncanonical cysteine (Fig. 1). By comparison, an IMGT analysis revealed that only 3 out of 55 (5%) of the human functional germline VH regions (IGHV2-70, IGHV4-4, and IGHV7-4-1) contain a noncanonical cysteine. Thus, cows have a significant preponderance of germline-encoded noncanonical cysteines, and because IGHV1-10 is used in ~70% of mature shorter CDR H3 HCs, VH regions with a noncanonical cysteine make up a very large proportion of the repertoire.

**FIGURE 2.**
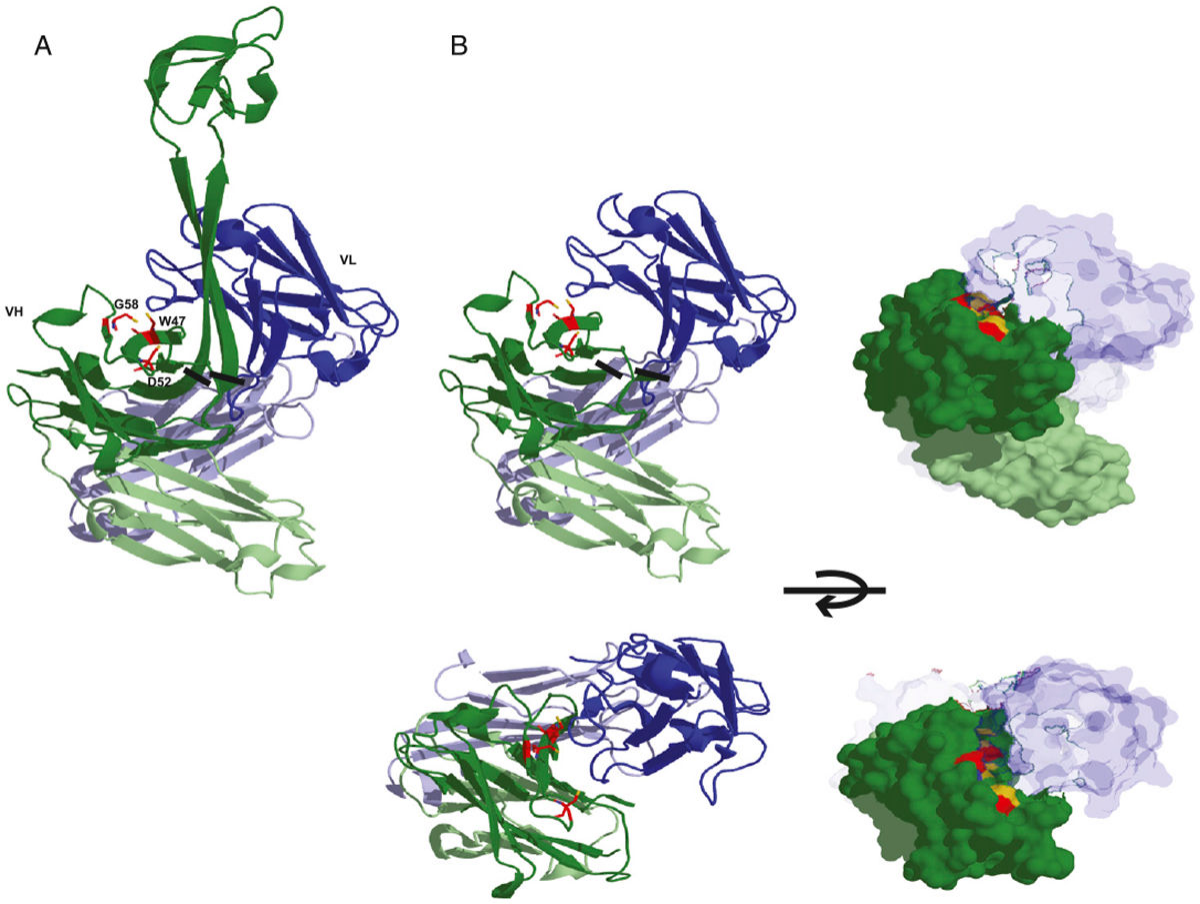
Modeling of cysteines in germline VHs reveals noncanonical cysteines in the paratope. BLV1H12 (PDB: 4K3D) was used as a model. (**A**) The Fab fragment of BLV1H12. The ultralong CDR H3 is bound by the black lines. The free cysteines highlighted in Fig. 1 are shown in red at their corresponding residues in the ribbon diagram of BLV1H12. (**B**) BLV1H12 without its CDR H3. Top, Ribbon (left) and space-filling (right) model of BLV1H12 without its CDR H3. Bottom, Top-down rotated view of BLV1H12 without its CDR H3, revealing the paratope with modeled cysteines. The HC is in green, and the LC is in blue. All figures were generated using PyMOL.

Because there are no crystal structures of shorter CDR H3 bovine Abs, we modeled the location of the unpaired cysteines using BLV1H12 (PDB identification: 4K3D), a bovine Ab with an ultralong CDR H3 (2), which uses IGHV1-7 (previously referred to as V_H_BUL, g1.110.20, IGHV1-1, IGHV10/30, and IGHV153). IGHV1-7 is highly homologous to IGHV1-10, IGHV1-21/IGHV1-33, and IGHV1-30, with 89, 88, and 86% identity at the amino acid level, respectively. These VH regions are frequently used in short CDR H3 Abs (5). In PyMOL, we substituted the BLV1H12 amino acids whose positions correspond to the noncanonical cysteines in IGHV1-10, IGHV1-21/IGHV1-33, and IGHV1-30 with cysteines (W47C, G58C, and D52C, respectively). This allowed us to observe that these cysteines are located directly across from CDR H3 and potentially in the Ag binding site, which is bounded by the two black lines in Fig. 2A We also deleted the ultralong CDR H3 to more easily visualize the location of the noncanonical cysteines in the VH region (Fig. 2B, top). In addition, we used a top-down view to visualize the location of these cysteines in relation to the rest of the HC and the LC (Fig. 2B, bottom). These models show that noncanonical cysteines in the VH region are in the putative Ag combining site and suggest that they could potentially form disulfide bonds with cysteines in CDR H3 or even the LC. Indeed, these unpaired cysteines come within 1.8 Å of the CDR L3 (20) and within 8.4 Å of the ultralong CDR H3 of BLV1H12. Importantly, whereas the β-ribbon stalk of the BLV1H12 CDR H3 extends far from the Ab surface, other CDR H3 regions with different structures may come much closer than 8.4 Å to the germline V region–encoded cysteines. Although it is very difficult to model the CDR H3, noncanonical cysteines found in germline bovine VH regions near CDR H2 could have the potential to disulfide bond with cysteines in the CDR H3 (Figs. 1, 2).

Because the majority of CDR H3 is encoded by the DH region, we analyzed all of the bovine germline DH regions for the presence of cysteines (Table I). Of the three possible forward DH reading frames, we analyzed the most hydrophilic DH reading frame (that contained the highest number of glycine and tyrosine residues) because these DH reading frames are known to be preferentially expressed in vertebrates (21), including cows (22). Remarkably, cysteines are found in 13 out of 16 DH regions (81%). Only three bovine germline DH regions are devoid of cysteines. Six DH regions (38%) contain one cysteine, six DH regions (38%) contain two cysteines, and one DH region (IGHD8-2, which is used to form ultralong CDR H3 Abs) contains four cysteines. Of the DH regions, 6 of 16 (38%) have an odd number of cysteines, and 7 of 16 (44%) have an even number of cysteines. From a diversity perspective, several DH regions are identical at the amino acid level (e.g., IGHD1-1, 1-2, and 1-4; IGHD3-1, 3-3, and 3-4; IGHD6-3 and 6-4; and IGHD9-1 and 9-4), so there are only 10 nonredundant DH regions, 8 of which encode cysteine. The DH regions have an abundance of Gly, Tyr, and Ser residues, and in many cases, the only differences between DH family members are the positions of cysteines (e.g., IGHD6-2 versus IGHD6-3). Thus, the bovine DH regions encode a significant diversity of cysteine residues, and, in some cases, cysteine position is the sole driver of diversity between segments.

### D region cysteines across species

**FIGURE 3.**
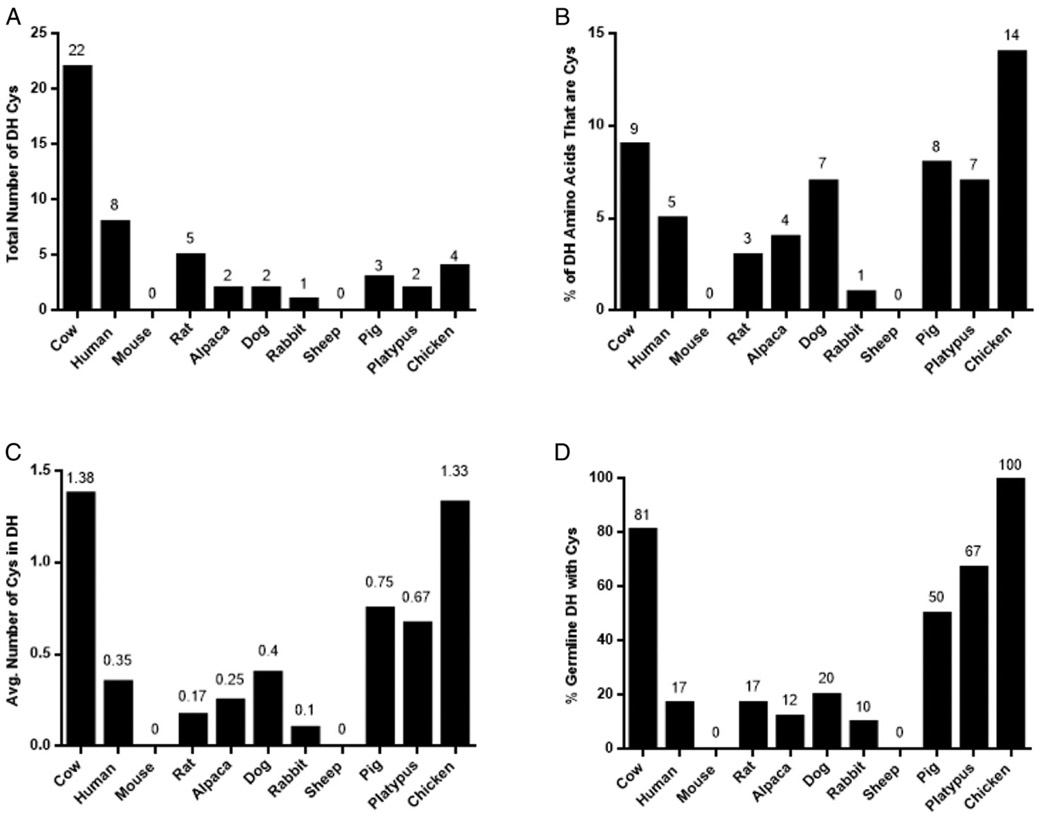
Cow germline DH regions have high cysteine content compared with other species. (**A**) The total number of cysteines in all DH regions combined is shown for 11 vertebrate species. (**B**) The percentage of cysteine content in all DH regions combined (total number of cysteines divided by total number of amino acids). (**C**) The average number of cysteines per DH region. (**D**) The percentage of germline DH genes that contain at least one cysteine. See Supplemental Tables I and II for gene sequences and underlying data.

Given the apparently high number of cysteines encoded in bovine germline DH regions, we compared cysteine DH content across several species (Fig. 3). When the total number of cysteines in DH regions are compared, cows are found to contain the most, with 22 cysteines in 16 DH regions, compared with 8 cysteines in 23 DH regions in humans, which is the next highest content (Fig. 3A, Supplemental Table I). Analysis of all DH region amino acids reveals that cysteines make up 9% of bovine DH region amino acids, compared with 5% in humans and 0% in mice and sheep but 14% in chickens, owing to the fact that chickens only have three DH regions, and all encode cysteine (Fig. 3B, Supplemental Table II). The average number of cysteines per DH region is highest in cows at 1.38, compared with 0.35 in humans, 0 in mice and sheep, and 1.33 in chickens (Fig. 3C). In contrast to cows, only 4 out of 23 (17%) human, 0 out of 25 (0%) mouse, 5 out of 29 (17%) rat, 1 out of 8 (12.5%) alpaca, 1 out of 5 (20%) dog, 1 out of 10 (10%) rabbit, and 0 out of 2 (0%) sheep germline DH regions contain a cysteine (Fig. 3D). Similarly to cows, 2 out of 4 (50%) pig, 2 out of 3 (67%) platypus, and 3 out of 3 (100%) chicken germline DH regions contain a cysteine. Thus, cows have the highest cysteine content in their germline DH repertoire, but other species might also use disulfide bonds in their repertoires.

### Cysteines in mature Abs

Because bovine germline VH and DH gene segments have a surprisingly high density of cysteines, we suspected that the mature Ab repertoire would also contain a high number of cysteines and that these residues, if found in even numbers, could form disulfide bonds. In this regard, an analysis of 10,869 mature bovine IgM and IgG HC sequences revealed that DH regions encoding an odd number of cysteines are most often recombined with VH regions encoding an odd cysteine (Table II). More than half of the sequences had an odd number of cysteines in both the VH and DH regions (6338 sequences), whereas another 24% of the sequences had an even number of cysteines in both the VH and DH regions (i.e., all cysteines were likely paired). By contrast, only 589 sequences (5%) had an odd cysteine in the DH region but no odd cysteine in the VH region, and 1288 sequences (12%) had an odd cysteine in the VH region but not the DH region. Only 2 out of 10,869HCs (0.0184%) contained only one cysteine in the VH region (Table II), which would indicate the loss of a canonical cysteine, which may be nonfunctional or the result of a sequencing error. Thus, when there is an even number of cysteines in the VH region, there is a 4.5 X likelihood to be an even number of cysteines in the DH region compared with an odd number. Likewise, when there is an odd number of cysteines in the VH region, it is five times as likely that there is an odd number of cysteines in the DH region compared with an even number. In other words, when there is a noncanonical cysteine in the VH region, there is often a cysteine in the DH region that it could pair with to form a disulfide bond.

**FIGURE 4.**
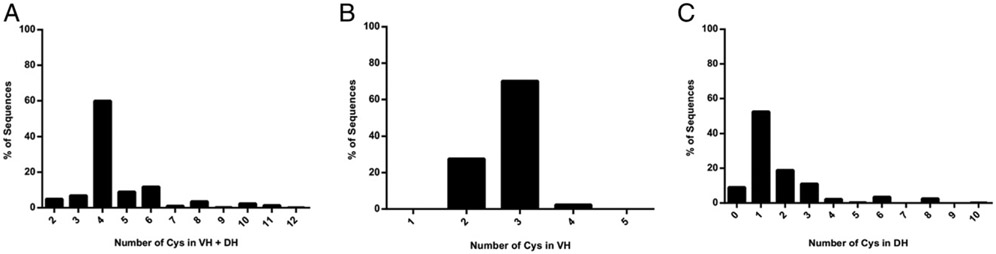
Bovine HCs are biased toward an even number of cysteines. Using a dataset of 10,869 Abs, the number of cysteines in (**A**) both the VH and DH regions, (**B**) VH alone, and (**C**) DH alone are tabulated.

Further analysis of these 10,869 VH genes revealed that the total number of cysteines in the VH region is an even number a majority of the time (84%) (Fig. 4A), with four cysteines (two noncanonical) being most common (60%). Remarkably, 95% of these sequences contained more than the two canonical cysteines in the V region. Parsing out the VH and DH regions allowed us to observe that the VH region most often has three cysteines (70%), whereas the DH region is most likely to have one cysteine (52%) (Fig. 4B, 4C). This analysis confirms that there is more likely to be an even number of cysteines in the VH region of bovine Abs than an odd number. When viewed in the context of Table II, these data support the notion that noncanonical cysteines in the VH region pair with cysteines in the DH region to form disulfide bonds.

### Role of SHM in cysteine creation

**FIGURE 5.**
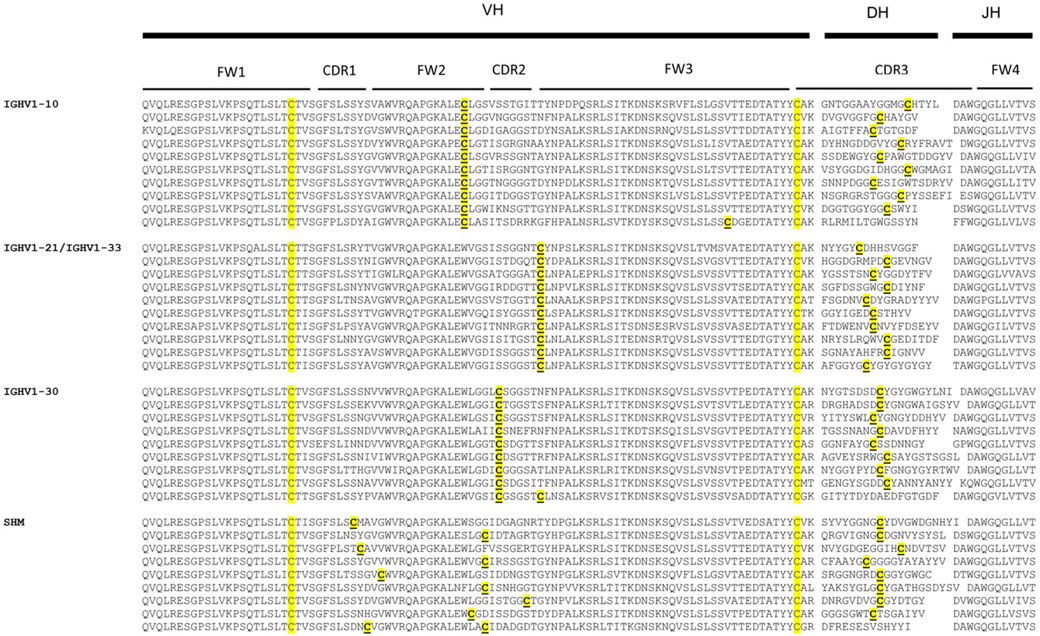
Mature bovine Ab sequences have noncanonical cysteines. Cysteines are highlighted in yellow, with noncanonical cysteines underlined in bold. The first and third cysteines in the VH region are conserved. The first 29 sequences contain germline-encoded unpaired cysteines, as found in IGHV1-10, IGHV1-21/IGHV1-33, and IGHV1-30 (see Fig. 1), whereas the last nine sequences contain unpaired cysteines that were generated by SHM. VH, DH, and JH regions are separated by a space.

Noncanonical cysteines in the HC can be 1) germline encoded in the VH or DH segments, 2) created through V(D)J recombination at the V-D or D-J junctions, or 3) introduced via SHM. The heavy mutation of the CDR H3 region as well as variations in junctional diversity preclude the assignment of CDR H3 cysteines as germline, junctional, or SHM derived. However, alignment of VH regions to their germline counterparts enable the identification of germline versus SHM-derived cysteines in the VH region. Fig. 5 shows an alignment of the V regions of 38 mature Abs from this dataset that contain two noncanonical cysteines in the V region. Twenty-nine of these Abs have germline-encoded noncanonical cysteines (see Fig. 1). In contrast, the bottom nine Abs have cysteines that are not germline encoded, which means that these cysteines have been introduced into the VH region by SHM. Surprisingly, 1402 out of 10,869 (13%) of the Abs in our dataset contain an odd cysteine in the VH region that is not found in the germline VH gene segment that was used, suggesting that mutation to cysteine in the VH region via SHM also significantly contributes to bovine Ab cysteine diversity. Presumably, the noncanonical cysteine in the VH region can form a disulfide bond with the odd cysteine encoded by the DH region. Also of interest are three unique sequences that have two noncanonical cysteines within the VH gene (see the last sequence of IGHV1-10, IGHV1-30, and SHM in Fig. 5), which apparently form a disulfide bond.

In previously studying the ultralong CDR H3 repertoire, we found that the ultralong IGHD8-2 gene, with repeating codons encoding Gly-Tyr-Gly, has an increased potential to mutate to cysteine (2, 11). In this regard, the codons GGT, AGT, and TAT encoding Gly, Ser, or Tyr can be mutated to TGT (encoding cysteine) with a single base change. These codons are not the most frequently used in the cattle genome, so they might have been selected for their potential to mutate to cysteine. Furthermore, the sequences 5′-GGTT-3′ and 5′-AGTT-3′ are affinity maturation “hot-spot” sequences as part of the DGYW/WRCH consensus recognition motif for AID (23). An analysis of the bovine germline DH regions reveals that most of these regions contain multiple AID hotspots (Table III). In fact, all 16 DH regions contain AID hotspots, ranging from 1 to 19 hotspots per region. Not surprisingly, IGHD8-2, which is the longest DH region and is used to form ultralong Abs, contains the most hotspots. The large number of AID hotspots in the bovine germline repertoire might allow for the facile formation of new cysteine codons in the DH region, which could be used to form new disulfide bonds in the resulting Ab.

### Cysteines in LC

Whereas the majority of HC genes have an even number of cysteines, several still have an odd number. Two possibilities exist to explain this result: 1) some or all of these cysteines may indeed be unpaired in the final Ab (24), or 2) the odd cysteines may disulfide bond with an odd number of cysteines in the LC. An alignment of germline l VL genes shows that 1 out of 25 (4%) VL genes contains an odd cysteine in CDR L1, allowing for the possible formation of disulfide bonds with unpaired cysteines in the HC (Supplemental Fig. 1). Additionally, upon sequencing 156 LC genes from a mature cow, we identified three mature bovine λ LCs with an unpaired cysteine in CDR L3 (Supplemental Fig. 1). Thus, it is possible that cow Abs contain unpaired cysteines, but they may also contain HC–LC interchain disulfide bonds as well.

## DISCUSSION

With limited germline-encoded V-D-J combinatorial potential compared with mouse and human, cows may use unconventional mechanisms for diversifying their primary Ab repertoire. We and others have previously shown that cysteines and disulfide bonds are important for creating diversity in the ultralong CDR H3 bovine Ab subset (2, 6, 11, 15, 25). In these ultralong Abs, multiple disulfide bonds in CDR H3 form different disulfide bond patterns and loop structures that contribute to structural diversity in a knob domain, which binds Ag (2, 6). A similar phenomenon could be happening in shorter CDR H3 bovine Abs as well, in which disulfide bonds between cysteines at different positions could create unique three-dimensional structures. Our analyses highlight the overall abundance of cysteines in bovine Abs and raise the possibility that disulfide bonds could be formed within CDRs, between CDRs, or between CDRs and FW.

Of particular interest, several cysteines are hard coded in bovine germline Ig gene segments in or near CDR H2. A free cysteine is encoded in FW2 of IGHV1-10, which is one of the most highly used VH regions in the bovine repertoire (5). Additionally, noncanonical cysteines are found in IGHV1-21/IGHV1-33 in FW3 and in IGHV1-30 in CDR H2. Modeling studies indicate that these cysteines are likely in the Ag combining site and could potentially pair with cysteines encoded by the DH region or even CDR L3. In this regard, cysteines are encoded in 81% of bovine germline DH regions, with 6 of 16 encoding a potentially pairable cysteine. Furthermore, codon biases in germline DH regions might facilitate an increased potential for mutating to cysteine during SHM. Thus, the germline capacity for cow Abs to combinatorially produce a repertoire with unique disulfide-bonded loops is significant.

The mature Ab repertoire has an abundance of cysteines and a diversity of potential disulfide bonds. Bovine Abs with an odd cysteine encoded by the VH region often also have an odd cysteine encoded by the DH region. Surprisingly, 95% of the mature HC sequences that we analyzed had additional noncanonical cysteines beyond the two completely conserved cysteines in the full V region. In addition to disulfide bonds in the HC, our analyses do not rule out that disulfide bonds could also be formed between the HC and LC. Indeed, one bovine germline λ VL region contains an unpaired cysteine. Furthermore, AID-mediated SHM could generate noncanonical cysteines in the CDRs of LCs, which would conceivably allow disulfide bonds to be formed between the V regions of bovine HCs and LCs. Supporting this hypothesis, we have discovered three mature bovine λ LCs with a noncanonical cysteine in CDR L3. Taken together, our findings indicate that disulfide bonds play an important role in most bovine Abs, likely contributing to diversity, stability, and Ag binding.

In addition to disulfide bonds created by V-D-J recombination of different gene segments, germline polymorphisms in VH, DH, and JH gene segments might also contribute to bovine Ab diversity. Multiple alleles are known to exist for some VH and JH genes, and recent work has uncovered multiple alleles for IGHD7-3 and IGHD8-2 (the DH region found in ultralong Abs) as well as possible new members of the IGHD8 family (26). Of note, the IGHD8-2 alleles encode cysteines at different positions, which may enable different disulfide-bonded loops to form. Along these lines, a cow that is heterozygous for VH, DH, and JH genes would have a greatly increased potential for Ab diversity compared with a homozygous cow.

Besides cows, camelids (camels, llamas, and alpacas) also contain Abs in which intrachain disulfide bonds play an important role. Camelids have a subset of Abs that consist of two HCs without any LC, termed camelid HC Abs (3, 27, 28). These Abs often contain CDR H3s that are longer, on average, than those found in mice and humans, with CDR H3 lengths of up to 24 aa. When these camelid HC Abs contain a cysteine in the CDR H3 loop, it often forms a disulfide bond with a cysteine in either CDR H1 or CDR H2, which serves to stabilize the relatively long CDR H3 (29-31). In chickens, cysteines in CDR H3 are thought to form disulfide bonds within CDR H3 and between CDR H3 and CDR H1 or CDR H2 (32). Because intrachain disulfide bonds contribute to Ab structure and diversity in camels, chickens, and cows, they could conceivably be important in other vertebrate species as well, including humans, whose CDR H3s range from 1 to 35 aa long, with 15 being average (33). In fact, 4 of the 23 (17%) functional human germline DH regions contain cysteines, allowing for the possible formation of intramolecular disulfide bonds within CDR H3. Through our analysis, we found that three germline human VH regions (IGHV2-70, IGHV4-4, and IGHV7-4-1) have an odd cysteine in either CDR H1 or FW3 that might play a role in intrastrand disulfide bond formation. Furthermore, in humans, amino acids on the Ab surface can be mutated to cysteine during SHM, leading to the formation of new disulfide bonds (34). In addition to cows, camels, chickens, and humans, intramolecular disulfide bonds might contribute to Ab structure and diversity in any mammalian species that has cysteines in its DH regions, such as rats, dogs, rabbits, pigs, and platypuses. Thus, disulfide bonds could play an important role in creating diversity not only in bovine Abs but in Abs from many vertebrate species.

## Supplementary Material

SupplementalInfo

## Figures and Tables

**TABLE I.** Bovine germline DH regions

Name	Sequence	Length (aa)	No. Cys
IGHD1-1	EYRDDGYCYT	10	1
IGHD1-2	EYRDDGYCYT	10	1
IGHD1-3	DYRDDGYCYT	10	1
IGHD1-4	EYRDDGYCYT	10	1
IGHD3-1	YCGSYCGSYYG	11	2
IGHD3-3	YCGSYCGSYYG	11	2
IGHD3-4	YCGSYCGSYYG	11	2
IGHD4-1	SYSGYGYGYSYGY	13	0
IGHD6-2	SCYSGYGYGCGYGYGYDY	18	2
IGHD6-3	SCYSGYGYGYGCGYGYGY	18	2
IGHD6-4	SCYSGYGYGYGCGYGYGY	18	2
IGHD7-3	SYGGYGYGGYGCYGYGYGYGY	21	1
IGHD7-4	SYGGYGYGGYGCYGYGYGYGYGY	23	1
IGHD8-2	SCPDGYSYGYGCGYGYGCSGYDCYGYGGYGGYGGYGYSSYSYSYTYEY	48	4
IGHD9-1	ELGG	4	0
	NSVG	4	0
	TRWG	4	0
IGHD9-4	ELGG	4	0
	NSVG	4	0
	TRWG	4	0

Cysteines are highlighted in yellow. DH regions with an odd number of cysteines have red cysteines, and DH regions with an even number of cysteines have green cysteines.

**TABLE II.** VH regions tend to pair with DH regions to yield a total number of cysteines that is even

VH Cys							
DH Cys	1	2	3	4	5	Odd	Even
0	0	536	431	20	0		
1	1	316	5222	154	5		
2	1	1268	728	54	1		
3	0	90	1082	17	2		
4	0	138	93	6	0		
5	0	7	26	1	0		
6	0	353	20	3	0		
7	0	3	0	0	0		
8	0	259	14	1	0		
9	0	1	0	0	0		
10	0	16	0	0	0		
Odd						6338	589
Even						1288	2654

Note that all VH regions have two completely conserved canonical cysteines.

**TABLE III.** There are abundant AID hotspots in bovine germline DH regions

Name	Sequence
IGDH1-1	AGAATACCGTGATGATGGTTACTGCTAGACC **EYRDDGYCYT**
IGDH1-2	AGAATATCGTGATGATGGTTACTGCTACACC **EYRDDGYCYT**
IGDH1-3	AGACTATCGTGATGATGGTTACTGCTACACC **DYRDDGYCYT**
IGDH1-4	AGAATATCGTGATGATGGTTACTGCTACACC **EYRDDGYCYT**
IGDH3-1	GTATTGTGGTAGCTATTGTGGTAGTTATTATGGTAC **YCGSYCGSYYG**
IGDH3-3	GTATTGTGGTAGCTATTGTGGTAGTTATTATGGTAC **YCGSYCGSYYG**
IGDH3-4	GTATTGTGGTAGCTATTGTGGTAGTTATTATGGTAC **YCGSYCGSYYG**
IGDH4-1	GTAGTTATAGTGGTTATGGTTATGGTTATAGTTATGGTTATAC **SYSGYGYGYSYGY**
IGDH6-2	GTAGTTGTTATAGTGGTTATGGTTATGGTTGTGGTTAT **SCYSGYGYGCGY** GGTTATGGTTATGATTATAC **GYGYDY**
IGDH6-3	GTAGTTGTTATAGTGGTTATGGTTATGGTTATGGTTGT **SCYSGYGYGYGC** GGTTATGGTTATAC **GYGYGY**
IGDH6-4	GTAGTTGTTATAGTGGTTATGGTTATGGTTGT**SCYSGYGYGYGC** GGTTATGGTTATGCTTATAC **GYGYGY**
IGDH7-3	GTAGTTATGGTGGTTATGGTTATGGTGGTTATGGTGTTAT **SYGGYGYGGYGCY** GGTTATGCTTATGGTTATGGTGGTTATGGTTGTTAT **GYGYGYGY**
IGDH7-4	GTAGTTATGGTGGTTATGGTTATGGTGGTTGTTAT**SYSSYSYSSYGCY** GGTTATGGTTATGGTTATGGTTATAC **GYGYGYGYGY**
IGDH8-2	GTAGTTGTCCTGATGGTTATAGTTATAGTTATGGTTATGGTTGT **SCPDGYSYGYGC** GGTTATGGTTATGGTTGTAGTGGTTATGATTGTTAT **GYGYGCSGYDCY** GGTTATGGTGGTTATGGTGGTTATGGTTATGCTTAT **GYGGYGGYGGYGY**AGTAGTTATAGTTATAGTTATACTTACGAATATAC **SSYSYSYTYEY**
IGDH9-1	GAACTCGGTGGGGC **ELGG** **NSVG** **TRWG**
IGDH9-4	GAACTCGGTGGGGC **ELGG** **NSVG** **TRWG**

Cysteines are highlighted in yellow. DH regions with an odd number of cysteines have red cysteines, and DH regions with an even number of cysteines have green cysteines. AID hotspots are boxed in red.

## Abbreviations used in this article:

AID: activation-induced cytidine deaminase
DH: HC D
FW: framework region
H2: HC 2
H3: HC 3
HC: H chain
IMGT: International Immunogenetics Information System
JH: HC J
LC: L chain
PDB: Protein Data Bank
SHM: somatic hypermutation
VH: HC V
VL: LC V
