# Supplementary material for: A Broad Role for Cysteines in Bovine Antibody Diversity
Source: Immunohorizons. Author manuscript; Available in PMC 2020 Jul 17. (PMC7366855; doi:10.4049/immunohorizons.1900058)
Supplement: SupplementalInfo [file NIHMS1605657-supplement-SupplementalInfo.pdf]

```
IGLV2   QAVLTQPSSVSGSLGQRVSITCSGSSNNIGSYGVGWYQQVPGSGLRTIIYGSSSRPSGVPDRFSGSKSGNTATLTISSLQAEDEADYFCATVDYSSSTV
IGLV6   QDVLTQPSSVSGSLGQNVSITCSGSSSNVGYANYVSWHQQKQGSAPRTLIYGATSRASGVPDQFSGSKSGNTATLTISSLQPEDEADYYCSSYDSSSNIG
IGLV8   QAVLTQPPSVSGSLGQTVTISCTGSSNNIGILGVSWYQQIPGSAPRTLIYNSNKRPSGVPDRFSGTKSGNTGTLTIASLQAEDEADYYCASADLSLTSP
IGLV10  QSGLTQPSSVSGNLGQTVITSCAGTSSYVGSYNGVGWYQQLPGSAPKTLIYNVSKRPSGIPDRFSGSKSGNTATLTVSGLQAEDEADYYCSSYKSGGSV
IGLV13  QSGLTQPSSVSGNLGQTVTISCAGTSSDVGAYNGVGWYQQLPGSAPKTLIYNLNKRSSGIPARFSGSKSGNTATLTISGLQAEDEADYYCSSYKSGGSV
IGLV14  SSQLTQPPAVSVSLGQTASITCQGDDLESYYAHWYQQKPSQAPVLVIYESSERPSGIPDRFSGSSSGNTATLTISGAQTEDEADYYCQSYDSSGDP
IGLV15  SYELTQPTSVSVALGQTAKITCSGDLLDEQYTQWYQQKPGQGPVRVIYKDSERPSGISDRFSGSSSGKTATLTISGAQTEDEADYYCQSADSSDNP
IGLV16  SYELTQLTSVSVALGQTAKITCSGELLDEQYTQWYQQKPGQAPKLVIYKDSKRRSGIPDQFSGSSSGKTAILTISGVRAEDEADYYCLSWDSGSYNV
IGLV17  SSQLTQPPAVSVSLGQTASITCQGDDLELLSAHWYQQKPGQAPVLVIYADDNLASGIPDRFSGSKSDTTATLTIRGAQAEDEADYYCQSADISGV
IGLV21  QPVLTQPVTVSASLGASARLSCTLSSGYNVSNYSIYWYQQKAGNPLRYLLRFKSDSDKHQGSGVPSRFSGSKDASTNAGLLLISGLQPEDEADYYCAVWHGDTNA
IGLV23  QAVLTQPPSVSGSLGQRVTISCTGSSSNIGGGNYVGWYQQIPGSAPKTLIYRSTSRPSGVPDRFSGSRSGNTATLTISSLQAEDEADYYCATYESSSYHG
IGLV25  QAVLTQPSSVSGSLGQRVSITCSGSSNNIGRYGVGWYQQVPGSGLRTIIYGSSSRPSGVPDRFSGSKSGNTATLTISSLQAEDEADYFCVAYDSSSSIV
IGLV27  QAVLTQPSSVSGSLGQRVSITCSGSSSNVGYGNYVSWFQEIPGSAPRTLIYGDTSRASGVPDRFSGSRSGNTATLTISSLQAEDEADYFCASYQSGNT
IGLV28  QAVLTQPSSVSGSLGQRVSITCSGSSSNVGTGNYVSWFQQIPGSAPRTLIYGATSRASGVPDRFSGSRSGNTATLTISSLQAEDEADYFCASYQSGNT
IGLV30  QAVLTQPSSVSGSLGQRVSITCSGSSSNVGNGYVSWYQLIPGSAPRTLIYGDTSRASGVPDRFSGSRSGNTATLTISSLQAEDEADYFCASAEDSSSNA
IGLV33  QAVLTQPSSVSGSLGQRVSITCSGSSSNVGTGNYVSWFQQIPGSAPRTLIYGATSRASGVPDRFSGSRSGNTATLTISSLQAEDEADYFCASYQSGNT
IGLV35  QAVLTQPSSVSGSLGQRVSITCSGSSNNIGRYGVGWYQQVPGSGLRTIIYGSSSRPSGVPDRFSGSKSGNTATLTISSLQAEDEADYFCATGDYSSSTV
IGLV37  QAVLTQPSSVSGSLGQRVSITCSGSSSNVGYGNYVSWFQEIPGSAPRTLIYGDTSRASGVPDRFSGSRSGNTATLTISSLQAEDEADYFCASYQSGNT
IGLV39  QAVLTQPSSVSGSLGQRVSITCSGSSNNIGSYGVGWYQQVPGSGLRTIIYGSSSRPSGVPDRFSGSKSGNTATLTISSLQAEDEADYFCAAGDSSSSTG
IGLV40  QTVIQEPSLSVSPGGTVTLTCGLSSGSVTTYNEPSWYRETPGQAPRNVIYNTNTPRTGVPDRFSASISGNKVTLTITGAQPEDEADYHCLLYQGSDSYG
IGLV43  QAVLTQPSSVSGSLGQRVSITCSGSSSNVGYGNYVSWFQQIPGSAPRMLIYGATSRASGVPDRFSGSRSGNTATLTISSLQAEDEADYFCASPDSSSSG
IGLV45  QAVLTQPPSVSGSLGQRVTISCTGSSSNIGGGNCVGWYQQIPGSAPKTLIYRSTSRPSGVPDRFSGSRSGNTATLTISSLQAEDEADYYCATYESSSYHG
IGLV46  QTVIQEPSLSVSPGGTVTLTCGLSSGSVTTYNEPSWYRETPGQAPRNVIYNTNTPRTGVPDRFSASISGNKVTLTITGAQPEDEADYLCLLYQGSDSYG
IGLV49  QAVLTQPSSVSGSLGQRVSITCSGSSSNVGLGNYVSWFQQIPGSAPRTLIYGATSRASGVPDRFSGSRSGNTATLTISSLQAEDEADYFCASPDSSSSS
IGLV55  QAVLTQPSSVSGSLGQRVSITCSGSSSNVGRGNYVNWFQQIPGSAPRTLIYGATSRASGVPDRFSGSRSGNTATLTISSLQAEDEADYFCAAGDSSSSTG

Mature  QAVLTQPSSVSGSLGQRVSITCSGSSNNIEKYNVGWYQQVPGSGLRTIIYATTNRPSGVPDRFSGSKSGNTATLTISSLQAEDEADYFCAAYDRSIKTAVCGSGTTLTVLGHRP
Mature  QAVVTQPSSVSGSLGQRVSITCSGNSRDVGNGYVSWYQLIPGSAPRTLIYDDTNRASGVPDRFSGSRSGNTATLTISSLQAEDEADYFCACTEDNSSAVFGSGSTLTVLGQPK
Mature  QAVLTQPSSVSGSLGQRVSITCSGSGSNIGSWYVGWYQQVPGSGLRTIIYGTRSRASGVPDRFSGSKSGNTATLTISSLQAEDEADYFCVVYDGASNCAVFGSGTTLTVLGQPK
```

Supplemental Figure 1

Supplemental Table I. Germline DH Sequences of 11 Mammalian Species

Species	DH	Sequence
Cow	DH1-1	E Y R D D G Y C Y T
Cow	DH1-2	E Y R D D G Y C Y T
Cow	DH1-3	D Y R D D G Y C Y T
Cow	DH1-4	E Y R D D G Y C Y T
Cow	DH3-1	Y C G S Y C G S Y Y G
Cow	DH3-3	Y C G S Y C G S Y Y G
Cow	DH3-4	Y C G S Y C G S Y Y G
Cow	DH4-1	S Y S G Y G Y G Y S Y G Y
Cow	DH6-2	S C Y S G Y G Y G C G Y G Y G Y D Y
Cow	DH6-3	S C Y S G Y G Y G Y G C G Y G Y G Y
Cow	DH6-4	S C Y S G Y G Y G Y G C G Y G Y G Y
Cow	DH7-3	S Y G G Y G Y G G Y G C Y G Y G Y G Y G Y
Cow	DH7-4	S Y G G Y G Y G G Y G C Y G Y G Y G Y G Y G Y
Cow	DH8-2	S C P D G Y S Y G Y G C G Y G Y G C S G Y D C Y G Y G G Y G G Y G G Y G Y S S Y S Y S Y T Y E Y
Cow	DH9-1	E L G G
Cow	DH9-4	E L G G
Human	DH1-1	G T T G T
Human	DH1-7	V L R F L E W L L Y
Human	DH1-20	G I T G T
Human	DH1-26	G I V G A T
Human	DH2-2	G Y C S S T S C Y A
Human	DH2-8	G Y C T N G V C Y T
Human	DH2-15	G Y C S G G S C Y S
Human	DH2-21	A Y C G G D C Y S
Human	DH3-3	V L R F L E W L L Y
Human	DH3-9	Y Y D I L T G Y Y N
Human	DH3-10	Y Y Y G S G S Y Y N
Human	DH3-16	Y Y D Y V W G S Y A Y T
Human	DH3-22	Y Y Y D S S G Y Y Y
Human	DH4-4	D Y S N Y
Human	DH4-17	D Y G D Y
Human	DH5-5	G Y S Y G Y

Human	DH5-12	G Y S G Y D Y
Human	DH5-18	G Y S Y G Y
Human	DH6-6	E Y S S S S
Human	DH6-13	G Y S S S W Y
Human	DH6-19	G Y S S G W Y
Human	DH6-25	G Y S S G Y
Human	DH7-27	L T G
Mouse	DH1-1	F I T T V V A
Mouse	DH1-2	F I T T A
Mouse	DH1-3	Y I T K V V A
Mouse	DH2-1	S T M V T
Mouse	DH2-2	S T M V T
Mouse	DH2-3	S M M V T
Mouse	DH2-4	S T M I T
Mouse	DH2-5	P T I V T
Mouse	DH2-6	P T I V T
Mouse	DH2-7	S T M V T
Mouse	DH2-8	S T M V T
Mouse	DH2-9	P T M V T
Mouse	DH2-10	P T M V T
Mouse	DH2-11	P S M V T
Mouse	DH2-12	Y Y S Y Y S Y D
Mouse	DH2-13	Y Y G D Y
Mouse	DH2-14	P T I G T
Mouse	DH3-1	G T A R A
Mouse	DH3-2	R Q L G L
Mouse	DH3-3	G T A R A
Mouse	DH4-1	L T G
Mouse	DH6-1	R Q L A S A V P Q
Mouse	DH6-2	R Q L V S A V P Q
Mouse	DH6-3	R Q L A S A V P Q
Mouse	DH6-4	R Q L A S A V P Q
Rat	DH1-1	Y Y Y S G D
Rat	DH1-2	Y Y Y S S Y I Y

Rat	DH1-3	F N Y G S Y
Rat	DH1-4	N Y P G I T
Rat	DH1-5	N N I G T T
Rat	DH1-6	F M Y T T D Y Y Y
Rat	DH1-7	S Y Y G Y D Y
Rat	DH1-8	F N Y S S Y
Rat	DH1-9	T Y Y G Y N Y
Rat	DH1-10	Y N N Y
Rat	DH1-11	L T T E G I V
Rat	DH1-12	Y Y Y D V V I I
Rat	DH2-1	R Y L
Rat	DH2-2	G Y L
Rat	DH2-5	G Y L
Rat	DH2-6	G Y L
Rat	DH2-7	G Y L
Rat	DH3-1	P L S A P T Q
Rat	DH3-2	P L Q C T H P
Rat	DH3-3	P L Q C P H P
Rat	DH3-4	P L Q C P Q P
Rat	DH3-5	P L Q C T H S
Rat	DH3-7	P L K C P Q P
Rat	DH4-1	D I I R A
Rat	DH4-2	G R I L E
Rat	DH4-3	G I I R G
Rat	DH4-4	G V I R G
Rat	DH4-5	G I I R G
Rat	DH5-1	L T G
Alpaca	DH1	Y Y A Y W L G D A
Alpaca	DH2	T Y Y S G S Y Y Y T
Alpaca	DH3	Y Y Y C S G Y G C Y D
Alpaca	DH4	Y Y S D Y D
Alpaca	DH5	R L R V G V
Alpaca	DH6	Y G S S W Y
Alpaca	DH7	E L V A P

Alpaca	DH8	L T G
Dog	DH1	Y Y C T D D Y C F N
Dog	DH2	Y Y G S Y Y
Dog	DH3	Y I Y M D
Dog	DH4	Y S S S W Y
Dog	DH6	L T G
Rabbit	DH1-1	A Y T S S S G Y Y I
Rabbit	DH2-1	S Y D D Y G D Y
Rabbit	DH3-2	P M G S W F P W L W G
Rabbit	DH3-3	P M G S L F P W L C S
Rabbit	DH4-1	Y Y S S G W G
Rabbit	DH4-2	Y A G S S W D
Rabbit	DH5-1	F G E G P T H H V A M V V
Rabbit	DH6-1	V T I V M V M L M L
Rabbit	DH7-1	Y P G Y S T G T
Rabbit	DH8-1	Y A G S S Y Y T
Sheep	DH2	S Y Y S G Y G Y A Y G Y
Sheep	DH3	D M I G M A V V T V V S L
Pig	DH1	N C Y S Y G A S C Y S D
Pig	DH2	D Y S G C Y S G Y
Pig	DH3	A E R T A L R P Q L A
Pig	DH4	L T G
Platypus	DH1	Y Y Y S S G C Y D
Platypus	DH2	Y Y Y S S G C Y D
Platypus	DH3	Y S Y G S S Y Y N Y
Chicken	DH1	G S G Y C G W G A Y
Chicken	DH2	G S A C C G P Y
Chicken	DH4	G R S A Y S C G A Y

Supplemental Table II. Summary of the Cysteine Content in the DH Regions of 11 Mammalian Species

Species	Number of Functional DH Regions	Total Number of Amino Acids	Total Number of Cysteines
Cow	16	240	22
Human	23	170	8
Mouse	25	146	0
Rat	29	165	5
Alpaca	8	56	2
Dog	5	30	2
Rabbit	10	95	1
Sheep	2	26	0
Pig	4	35	3
Platypus	3	28	2
Chicken	3	28	4
